# HIV-induced matrix metalloproteinase-9 activation through mitogen-activated protein kinase signalling promotes HSV-1 cell-to-cell spread in oral epithelial cells

**DOI:** 10.1099/jgv.0.001075

**Published:** 2018-05-18

**Authors:** Irna Sufiawati, Sharof M. Tugizov

**Affiliations:** ^1^​ Department of Oral Medicine, Faculty of Dentistry, Universitas Padjadjaran, Bandung, Indonesia; ^2^​ Department of Medicine and Department of Orofacial Sciences, University of California, San Francisco, CA, USA

**Keywords:** HIV, HSV-1, MAPK, matrix metalloproteinase-9, NF-κB

## Abstract

We have shown that cell-free HIV-1 and viral proteins tat and gp120 activate mitogen-activated protein kinases (MAPKs) in tonsil epithelial cells, disrupting their tight and adherens junctions. This causes liberation of the HSV-1 receptor nectin-1 from assembled adherens junctions, leading to promotion of HSV-1 infection and spread. In the present study, we show that HIV-associated activation of MAPK leads to upregulation of transcription factor NF-κB and matrix metalloproteinase-9 (MMP-9). This induces the disruption of tight and adherens junctions, increasing HSV-1 cell-to-cell spread. Inhibition of HIV-associated MAPK activation by U0126 abolishes NF-κB and MMP-9 upregulation and reduces HSV-1 spread. Inactivation of MMP-9 also reduced HIV-promoted HSV-1 spread. These results indicate that HIV-1-activated MAPK/NF-κB and MMP-9 play a critical role in the disruption of oral epithelial junctions and HSV-1 cell-to-cell spread. Inhibition of MMP-9 expression in the oral epithelium of HIV-infected individuals may prevent the development of diseases caused by HSV-1, such as ulcers, necrotic lesions and gingivostomatitis.

## Introduction

Persistent and shedding oropharyngeal mucosal ulcers associated with herpes simplex virus (HSV) type 1 infection have been reported in human immunodeficiency virus (HIV)-infected patients [1, 2]. HIV-associated dysfunction of both the adaptive and innate immune responses against HSV leads to increased frequency of its reactivation and spread in HIV-infected patients [3, 4]. Reactivation and shedding of HSV is also observed in HIV-infected patients treated with antiretroviral therapy [5, 6]. Although HIV-associated immune dysfunction is important in regard to HSV infection and spread, the possible molecular interaction between HSV and HIV may also play a critical role. Our previous study showed that HIV-1-activated, mitogen-activated protein kinase (MAPK) signalling induced the disruption of tight and adherens junctions of polarized oral epithelial cells, liberating nectin-1, an essential receptor for HSV-1 envelope glycoprotein D (gD) [7]. Furthermore, binding of HSV-1 gD to nectin-1 facilitated HSV-1 infection and cell-to-cell spread [7].

Two HIV proteins independently activate MAPK: HIV tat is a transactivator protein that binds to α5β1, α5β3, and αvβ3 integrins [8–11] and induces ras-dependent activation of MAPK [12]. HIV-1 envelope glycoprotein gp120 binds to the galactosylceramide (GalCer) and chemokine receptors CCR5 and/or CXCR4, inducing MAPK activation [13–16]. We and others have shown that oral and genital epithelial cells express β1 and αV integrins. GalCer, CCR5 and/or CXCR4 [17–28], and HIV tat and HIV gp120 induce activation of MAPK [7].

MAPK-associated activation of NF-κB signalling may upregulate matrix metalloproteinase-9 (MMP-9) expression [29–31]. Furthermore, overexpression of MMP-9 is associated with disruption of tight and adherens junctions [32, 33]. MMP-9 interaction with tight and adherens junctions proteins occludin and E-cadherin, respectively, leads to their proteolytic degradation [34–37], resulting in a loss of epithelial junction integrity.

In this work, we investigated the role of HIV-activated MAPK and NF-κB signalling in MMP-9 activation and its part in disrupting epithelial junctions and promoting HSV-1 spread within polarized tonsil epithelial cells. We show that cell-free HIV-1 virions and proteins tat and gp120 induce activation of MAPK/NF-κB pathways, which induce MMP-9 expression. Furthermore, HIV-activated MMP-9 leads to the disruption of tight and adherens junctions, thus promoting HSV-1 cell-to-cell spread.

## Results

### HIV-1 virions and viral tat and gp120 proteins activate MAPK/ERK1/2 and NF-κB signalling pathways in polarized tonsil epithelial cells

We have shown that cell-free HIV-1_SF33_ virions and proteins tat and gp120 induce activation of the extracellular signal-regulated kinases (ERK)/MAPK signalling pathway [7]. We investigated whether HIV-1-activated MAPK leads to the activation of NF-κB signalling in tonsil epithelial cells. Polarized tonsil epithelial cells were exposed to dual-tropic (X4 and R5) cell-free HIV-1_SF33_ virions and recombinant tat and gp120 in combination for 5 days with or without the MAPK inhibitor U0126. Mutant inactive tat lacking its basic domain and arginine-glycine-aspartate (RGD) motif and heat-inactivated gp120 were used as negative controls [7]. Treatment of cells with virus and its proteins and MAPK inhibitor U0126 did not cause any toxic effect in the cells (data not shown).

**Fig. 1.**
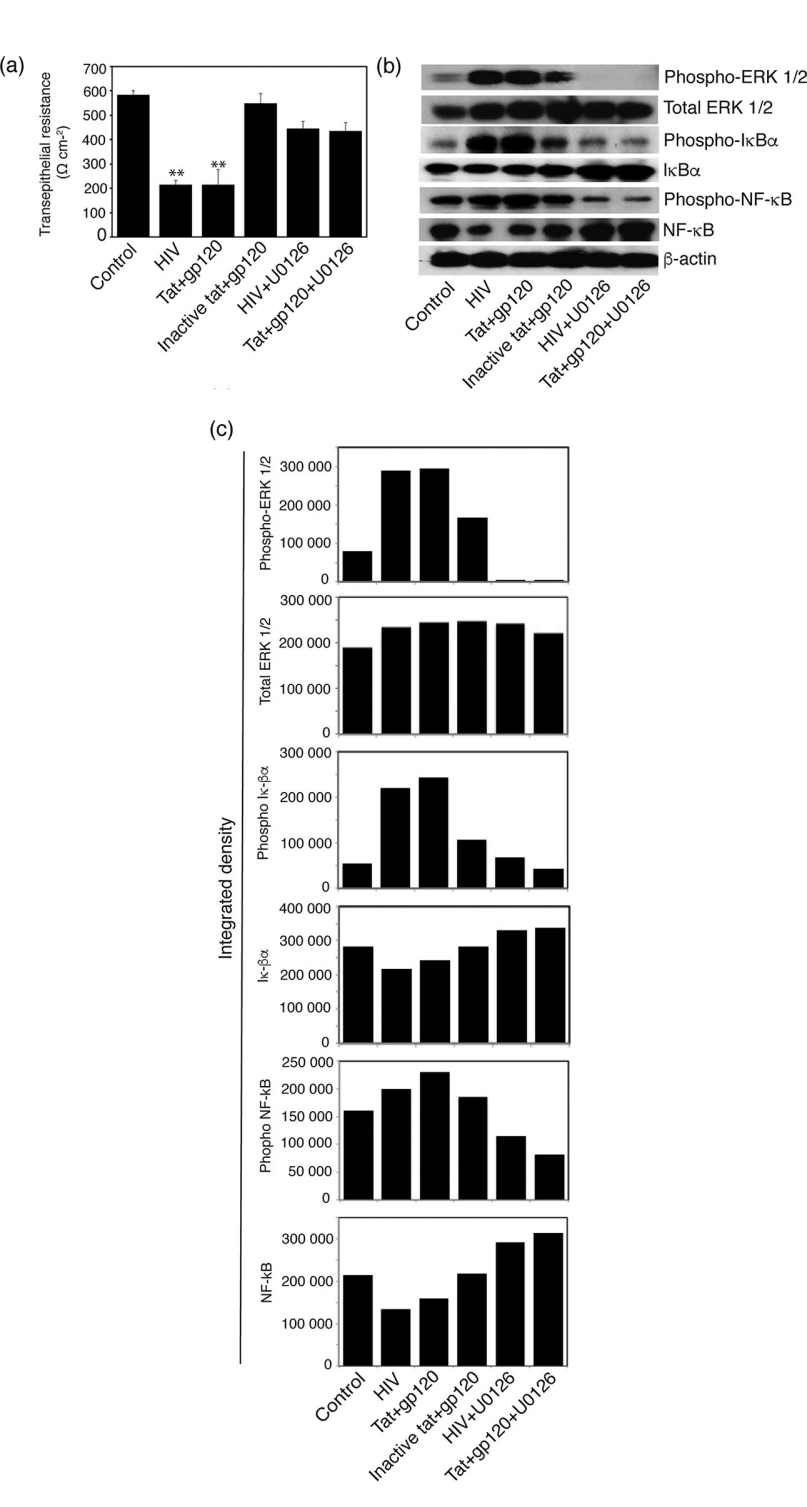
HIV-1-induced activation of MAPK/ERK1/2 upregulated NF-κB signalling in polarized oral epithelial cells. (a) Polarized tonsil epithelial cells were exposed to cell-free HIV_SF33_ virions and active and inactive tat and gp120 proteins in combination, in the absence or presence of MEK1/inhibitor U0126 for 5 days. Untreated cells were used as a control. TER of polarized cells was then measured on day 5. Error bars indicate sem (*n*=3). **P<0.001, compared to the control. (b) After measurement of TER, cells were collected and used to examine the expression of phosphorylated and total ERK1/2 using Western blot assay. The same cell samples were examined for phosphorylation and degradation of IκBα and NF-κB p65. (c) To quantify phosphorylation of ERK1/2, IκBa, and NF-κB, we measured the integrated densities of pixels in the protein bands from panel (b) by ImageJ software and present the data as a bar graph. Two independent experiments showed similar results.

To examine the disruption of tight junctions of polarized epithelial cells, their trans-epithelial resistance (TER) was measured with an epithelial voltohmmeter. We have shown that reduction of TER indicates disruption of tight and adherens junctions [7]. TER was reduced in cells treated with HIV-1_SF33_ virions and active tat+gp120 by ~60 % compared to control untreated cells and cells treated with inactive tat+gp120. In the presence of the MAPK inhibitor, the reduction of TER by HIV-1 virions and tat+gp120 proteins was not significant (Fig. 1a).

These cells were then used to examine MAPK and NF-κB activation by Western blot assay (Fig. 1b); the protein bands were quantified using ImageJ software (Fig. 1c). Exposure of polarized tonsil epithelial cells to HIV-1_SF33_ virions and tat+gp120 led to the induction of MAPK/ERK1/2 phosphorylation (Fig. 1b, c). In addition, we found that HIV-1 virions and proteins increased IκBα and NF-κB phosphorylation (Fig. 1b, c). It is well known that inducible NF-κB activation is initiated by phosphorylation and degradation of IκBα protein, which leads to NF-κB translocation to the nucleus, where it binds DNA and induces transcription of target genes [38, 39]. In the presence of the MAPK inhibitor U0126, HIV-1- and tat+gp120-induced phosphorylation of MAPK/ERK1/2, IκBα and NF-κB was abolished (Fig. 1b, c). These data indicate that HIV-1- and tat+gp120-induced activation of MAPK/ERK1/2 leads to the activation of NF-κB in polarized oral epithelial cells.

### HIV-1 virions and tat+gp120 proteins induce MMP-9 expression through MAPK/NF-κB signalling in polarized oral epithelial cells

**Fig. 2.**
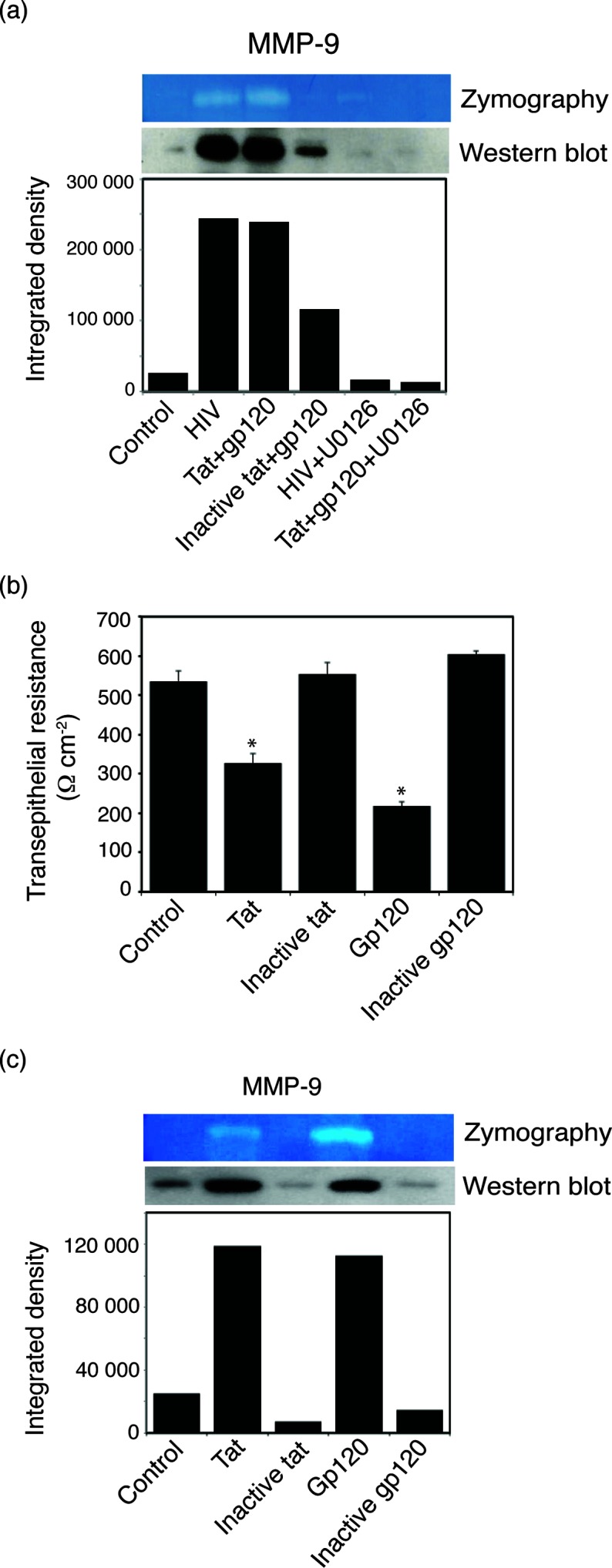
HIV-1 virions and proteins tat and gp120 enhanced MMP-9 expression and its activity in polarized oral epithelial cells. (a) Polarized tonsil epithelial cells treated with HIV-1_SF33_ virions and active and inactive tat+gp120 in the absence or presence of MAPK inhibitor U0126 for 5 days. The cells and culture medium were separately examined for expression and activity of MMP-9, respectively. (b) Polarized epithelial cells were separately treated with active and inactive tat, and and TER was measured on day 5. Error bars indicate sem (*n*=3). *P<0.001, compared to the control. (c) Cells were separately treated with active and inactive tat and gp120. Expression and activity of MMP-9 were examined in the cells and culture medium, respectively. (a and c) The integrated densities of pixels in the protein band were measured by ImageJ software and are presented as a bar graph. Two independent experiments showed similar results.

To determine whether the HIV- and tat+gp120-mediated activation of MAPK and NF-κB signalling pathways leads to the induction of MMP-9 expression and activity, polarized tonsil epithelial cells were incubated with HIV-1_SF33_ virions and tat+gp120 proteins with or without the MAPK inhibitor U0126. Untreated cells and cells treated with inactive tat+gp120 served as controls. Cells were examined for MMP-9 expression by Western blot assay, and culture medium was tested for activation of MMP-9 using gelatine zymography. The results showed that treatment of cells with HIV-1 virions and tat+gp120 increased MMP-9 expression by seven- to eightfold compared to untreated controls (Fig. 2a). Inactive tat+gp120 also showed about a threefold increase of MMP-9 compared to untreated controls. However, an active form of MMP-9 was detected only by HIV-1 virions and tat+gp120. Treatment of cells with the MAPK inhibitor U0126 abolished HIV- and tat+gp120-induced expression and activation of MMP-9 (Fig. 2a).

Next, we studied the independent roles of tat and gp120 proteins in reducing TER of polarized cells and inducing expression and activity of MMP-9. Cells were separately treated with active and inactive tat or gp120. The TER of polarized cells was decreased by ~40 and 60 % by active tat and gp120, respectively (Fig. 2b). In contrast, when the cells were treated with inactive tat and gp120, TER was not reduced compared to untreated controls. Analysis of MMP-9 expression and activity in cells treated separately with tat and gp120 showed that both HIV proteins induced MMP-9 expression and activity compared to inactive forms of tat and gp120 (Fig. 2c). Although induction of MMP-9 expression by tat and gp120 was similar, gp120-induced activity of MMP-9 was higher than tat-induced activity.

### HIV-induced MAPK/NF-κΒ and MMP-9 activation leads to the disruption of tight and adherens junctions in polarized oral epithelial cells

**Fig. 3.**
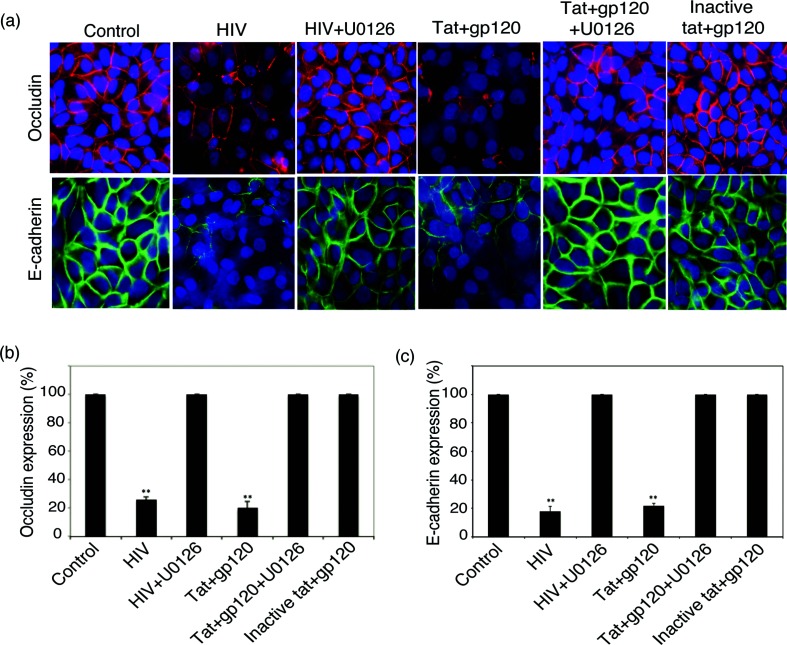
Disruption of tight and adherens junctions of oral epithelial cells induced by HIV-1 virions and tat and gp120. (a) Polarized tonsil epithelial cells were treated with HIV-1_SF33_ virions and active and inactive tat+gp120 proteins in the absence or presence of the MAPK inhibitor U0126. On day 5, cells were fixed and immunostained for occludin (red) and E-cadherin (green) and analyzed by fluorescence microscopy. Cell nuclei were stained blue. (b and c) Cells lacking expression of occludin or E-cadherin were counted in at least 10 fields, and their average numbers in three independent experiments are presented as the percentage of cells lacking occludin or E-cadherin expression. Error bars indicate sem (*n*=3).

To study the role of HIV-activated MAPK/NF-κB and MMP-9 in the disruption of tight and adherens junctions of polarized tonsil epithelial cells, we added HIV-1_SF33_ virions and tat+gp120 proteins to the cells with or without the MAPK inhibitor U0126. On day 5, cells were examined by immunofluorescence microscopy for expression of the tight junction protein occludin and the adherens junction protein E-cadherin by. In all control cells, occludin and E-cadherin were expressed in the membranes in a ring shape, which is the typical localization for intact tight and adherens junctions (Fig. 3a). In contrast, in cells treated with HIV-1 virions and active tat/gp120, expression of occludin and E-cadherin was partially or completely lost. Quantitative analysis showed that HIV-1 virions and active tat+gp120 reduced the expression of occludin and E-cadherin in ~70–80 % of cells (Fig. 3b, c). In contrast, expression of occludin and E-cadherin in cells treated with inactive tat+gp120 was detected in most of the cells (90–100 %), and proteins were localized in the membranes as a ring shape, indicating intact tight and adherens junctions. MAPK inhibitor U0126 reduced HIV-1- and active tat+gp120-induced disruption of tight and adherens junctions in ~90 % of cells, confirming the critical role of HIV-induced activation of MAPK/NF-κB/MMP-9 in the disruption of epithelial junctions.

### HIV-1 activates MAPK/NF-κB/MMP-9-promoted HSV-1 cell-to-cell spread in polarized oral epithelial cells

**Fig. 4.**
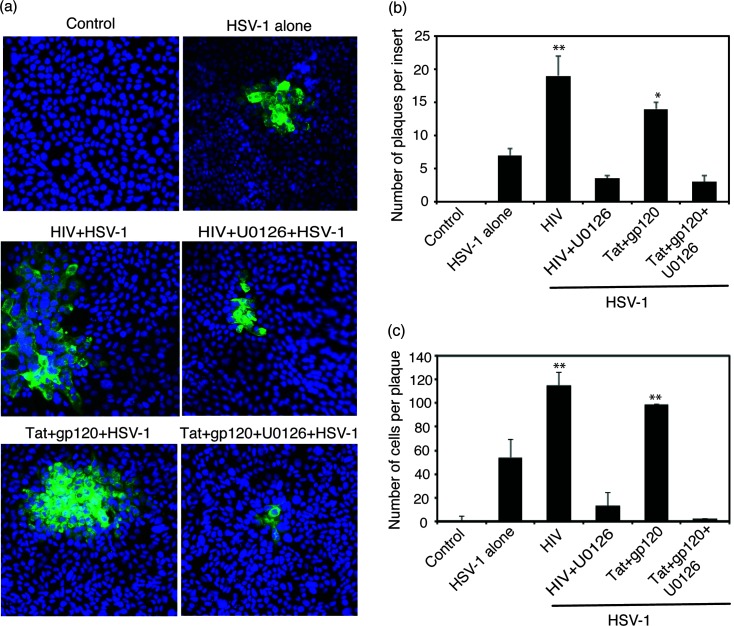
HIV-induced activation of MAPK/NF-κB and MMP-9 facilitated cell-to cell spread of HSV-1. (a) Polarized tonsil epithelial cells were exposed to cell-free HIV-1_SF33_ virions and tat+gp120 in combination for 5 days, with or without U0126. On day 5, epithelial cells were infected with HSV-1 from the basolateral surface and incubated for 3 days. Cells were fixed and then immunostained for HSV gD (green). Cell nuclei were stained blue. (b) HSV-1-infected plaques were counted on three inserts for each experiment. Results are presented as the average number of plaques or average number of infected cells per plaque. (c) HSV-1-infected cells were quantified in 10 random microscopic fields per plaque. Error bars indicate sem (*n*=3). *P<0.005, **P<0.001, all compared to the control group. Two independent experiments showed similar data.

Our previous study showed that HIV virion- and tat+gp120-induced disruption of adherens junctions liberated sequestered nectin-1, which facilitates HSV-1 spread because nectin-1 is a receptor for HSV-1 gD [7]. Here we investigated whether HIV-induced MAPK/NF-κB/MMP-9 activation and disruption of tight and adherens junctions of tonsil epithelial cells promotes cell-to-cell spread of HSV-1. For these experiments, we exposed polarized tonsil epithelial cells to HIV-1_SF33_ virions and tat+gp120 proteins, with or without U0126, for 5 days. Cells were then infected with HSV-1 from basolateral membranes; after 3 days cell-to-cell spread of virus was examined for plaque (foci) development by immunofluorescence microscopy. Quantitative analysis of plaque numbers showed about threefold more HSV plaques in cells treated with HIV-1 and tat+gp120, compared to control cells without HIV and tat+gp120 proteins (Fig. 4a, b). MAPK inhibitor U0126 reduced HSV-1 plaque numbers by three- to fourfold.

We also evaluated the average number of HSV-infected plaques. The results showed that the number of HSV-1-infected cells per plaque not treated with HIV-1 and tat+gp120 was ~40–50 (Fig. 4a, c). Pre-incubation of cells with HIV-1 and tat+gp120 increased the number of HSV-infected cells per plaque by more than twofold. The MAPK inhibitor U0126 reduced HSV-1 plaque size by ~10- to 12-fold.

These data revealed that HIV-induced MAPK/NF-κB/MMP-9 activation leads to an increase in HSV-1 cell-to-cell spread due to disruption of epithelial junctions and liberation of nectin-1. Reduction of HSV spread by U0126 indicates that blocking of upstream MAPK signalling may downregulate NF-κB-induced activation of MMP-9 and therefore reduce the HIV-facilitated disruption of epithelial junctions and cell-to-cell spread of HSV-1 in tonsil epithelial cells.

### Analysis of MAPK/NF-κB signalling and MMP-9 activation in polarized tonsil epithelial cells exposed to dual-tropic HIV-1_SF33_, R5-tropic HIV-1_SF170_ and X4 tropic HIV-1_92UG029_

**Fig. 5.**
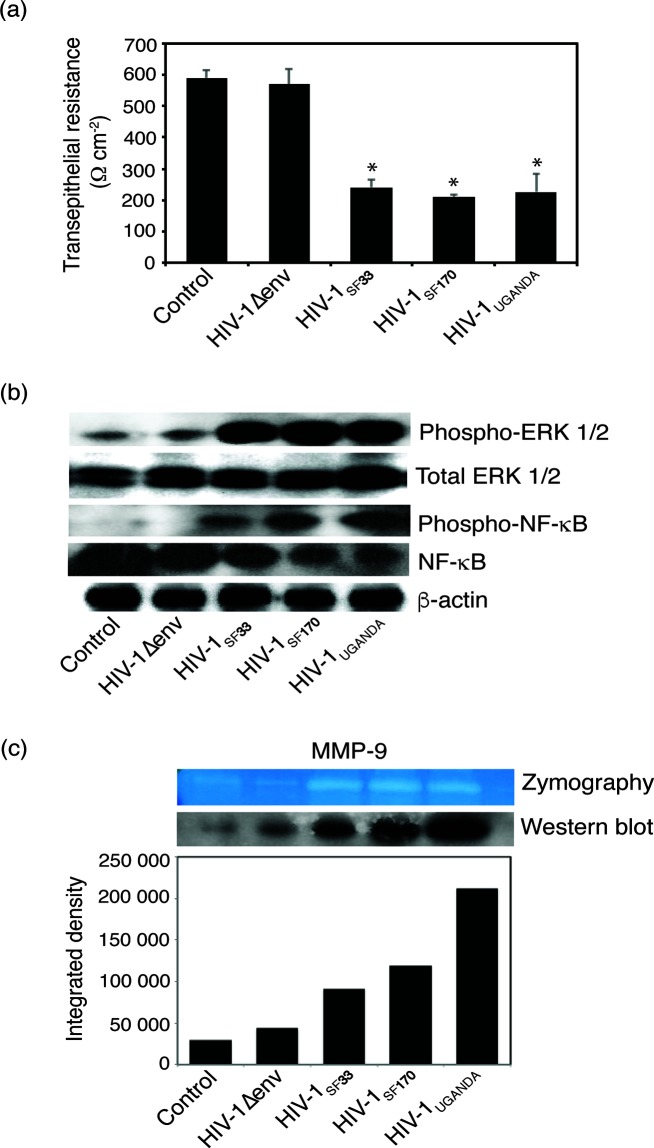
Activation of MAPK/NF-κB/MMP-9 by dual-tropic HIV-1_SF33_, R5-tropic HIV-1_SF170_ and X4-tropic HIV-1_92UG029_ in polarized tonsil epithelial cells. (a) Polarized tonsil epithelial cells were exposed to dual-tropic HIV-1_SF33_, R5-tropic HIV-1_SF170_ or X4-tropic HIV-1_92UG029_ (Uganda) for 5 days. Untreated cells and HIV-1∆env were used as controls. The TER of polarized cells was measured on day 5. Error bars indicate sem (*n*=3). *P<0.001, compared to the control group. (b) The cells were dissociated from inserts and used for evaluation of MAPK/ERK1/2 and NF-κB activation by Western blot assay. (c) The culture medium was collected and examined for MMP-9 expression and activity by Western blot and gelatine zymograpy, respectively. Two independent experiments showed similar outcome.

We next examined the activation of MAPK/NF-κB pathways and MMP-9 expression and activity in tonsil epithelial cells exposed to dual-tropic HIV-1_SF33_, R5-tropic HIV-1_SF170_ and X4-tropic HIV-1_92UG029_. Polarized oral epithelial cells were exposed to these three HIV-1 strains for 5 days. Controls included untreated cells and cells exposed to virus lacking HIV-1 envelope gp120 (HIV-1∆env). Analysis of TER of polarized cells showed that all three strains of HIV-1 reduced the TER by ~60–70 % compared to untreated controls (Fig. 5a). Reduction of TER by HIV-1∆env was not significant.

A Western blot assay was performed to determine the activation of MAPK/ERK1/2 and NF-κB and MMP-9 expression in tonsil epithelial cells by dual-tropic HIV-1_SF33_, R5-tropic HIV-1_SF170_ and X4-tropic HIV-1_92UG029_. We found that exposure of cells to all three strains of HIV-1 led to the activation of MAPK/ERK1/2 and NF-κB (Fig. 5b). In contrast, activation of MAPK/ERK1/2 and NF-κB was not detected by HIV-1∆env. We also found increased expression and activity of MMP-9 in cells treated with the three strains of HIV-1. Only slight expression of MMP-9 was observed in cells treated with HIV-1∆env (Fig. 5c). These results indicate that activation of MAPK/ERK1/2/NF-κB/MMP-9 in tonsil epithelial cells by HIV-1 is not dependent on viral tropism – i.e. dual-tropic HIV-1_SF33_, R5-tropic HIV-1_SF170_ and X4-tropic HIV-1_92UG029_ strains may lead to the disruption of epithelial junctions and the liberation of nectin-1, promoting HSV-1 spread.

### Inhibition of MMP-9 activation abolished HIV- and tat- and/or gp120-induced disruption of epithelial junctions and reduced HSV-1 spread

**Fig. 6.**
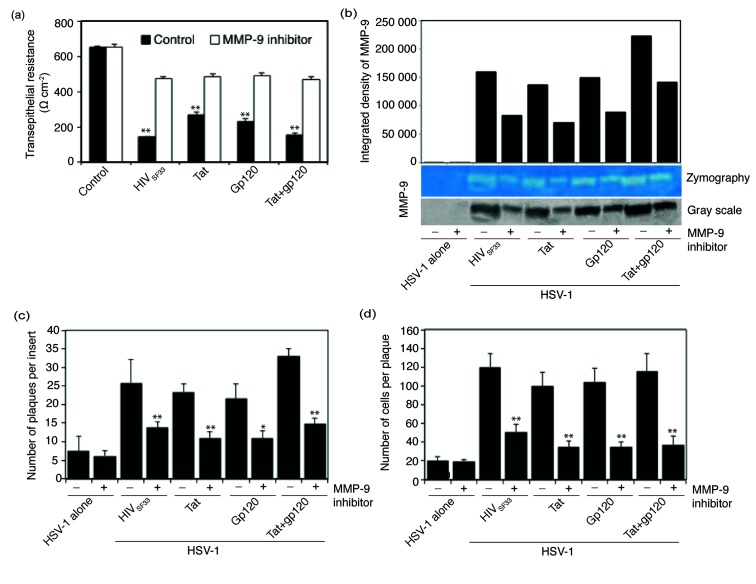
HIV-induced activation of MMP-9 is directly involved in cell-to-cell spread of HSV-1. (a and b) Polarized tonsil epithelial cells were exposed to cell-free HIV-1_SF33_ virions and tat and/or gp120 for 5 days in the presence of MMP-9 inhibitor I. Control cells were treated with DMSO at the same concentration as MMP-9 inhibitor I in treated cells. On day 5 the TER of polarized cells was measured (a). The culture medium of cells from panel (a) was collected and examined for MMP-9 activity by gelatine zymograpy. For quantitative analysis, gelatine zymograpy was converted to greyscale mode and the integrated density of protein bands was measured using ImageJ software (b). (c and d) Polarized epithelial cells exposed to HIV and tat and/or gp120 were infected with HSV-1 from the basolateral surface and incubated for 3 days in the presence or absence of MMP-9. Results are presented as the average number of plaques per insert (c) or the average number of infected cells per plaque (d). Error bars indicate sem (*n*=3). *P<0.005, **P<0.001, all compared to the control group. Two independent experiments showed similar results.

To examine the direct role of HIV- and tat/gp120-induced activation of MMP-9 in the disruption of epithelial junctions and HSV spread, we exposed polarized tonsil epithelial cells to HIV virions and tat and/or gp120 proteins in the presence or absence of MMP-9 inhibitor. Measurement of TER (Fig. 6a) and MMP-9 activity (Fig. 6b) in these cells showed that MMP-9 inhibition led to the reduction of TER induced by HIV and tat- and/or gp120 approximately two- to threefold compared to untreated controls (Fig. 6a, b). Thus, inactivation of MMP-9 in the cells exposed to HIV virions and tat and/or gp120 proteins abolished HIV- and tat- and/or gp120-induced disruption of epithelial junctions. Next, we examined HSV spread in cells exposed to HIV virions and tat and/or gp120 proteins in the presence or absence of MMP-9 inhibitor. Quantitative analysis of HSV-1-infected plaques showed that the inhibition of MMP-9 reduced plaque numbers of cells exposed to HIV or its proteins by 50–60 % compared to control cells (Fig. 6c). Analysis of the average size of HSV-infected plaques in cells exposed to HIV virions and tat and/or gp120 proteins also showed that inactivation of MMP-9 reduced HSV-1 spread facilitated by HIV virions or its proteins (Fig. 6d). These data clearly show that HIV-associated activation of MMP-9 plays a direct role in HSV-1 spread through the disruption of epithelial junctions.

## Discussion

Recently, we showed that HIV-1 virions and proteins tat and gp120 activate MAPK in tonsil epithelial cells, leading to disruption of their tight and adherens junctions and liberation of nectin-1, thus facilitating HSV-1 infection and spread. In the present study, we have shown that HIV-1-induced activation of MAPK led to upregulation of NF-κB signalling and MMP-9, which disrupted tight and adherens junctions of tonsil epithelial cells and facilitated HSV-1 cell-to-cell spread.

The signalling cascade of MAPK, including the ERKs (ERK1/2 or p42/p44), the c-Jun N-terminal kinases (JNKs) (JNK/SAPK) and the p38 MAPK, leads to downstream activation of NF-κB [40, 41]. NF-κB is a family of dimeric transcription factors (RelA/P65:P50) involved in multiple events including inflammatory and immune responses to infection. In unstimulated cells, the dimers of NF-κB are sequestered in the cytoplasm by a family of inhibitors (IκB proteins). In stimulated cells, the activation of NF-κB causes phosphorylation of IκB, followed by its ubiquitination and subsequent degradation [40, 41]. It is well documented that the activation of NF-κB, through the liberation of functional NF-κB dimers from IκB, allows its translocation from the cytoplasm into the nucleus, leading to upregulation of the expression of target genes [42], including MMP-9 [39, 43]. It is also possible that MAPK-activated tumour necrosis factor alpha (TNF-α) may activate MMP-9 [29, 44] – i.e. HIV-induced MAPK activation may upregulate MMP-9 through the activation of both NF-κB and TNF-α.

In normal oral mucosa epithelium, MMP-9 expression is negative or weak [45–48]. Our findings indicate that the interaction of HIV and its proteins tat and gp120 substantially induces the expression of MMP-9 through MAPK/NF-κB signalling, which leads to the disruption of epithelial junctions and HSV spread. To the best of our knowledge, we have shown for the first time that HIV-activated MMP-9 may facilitate the spread of HSV, which is an opportunistic infection for HIV/AIDS. Furthermore, our results suggest that inhibition of the MAPK/ERK1/2 signalling pathway can prevent the activation of MMP-9 and reduce HSV spread. Thus, inhibition of MAPK, NF-κB and MMP-9 in the oral cavity of HIV-infected individuals may lead to the reduction or elimination of HSV-induced lesions and ulcerations.

MMP-9 has been shown to disrupt tight junctions by cleaving claudin-1, occludin and ZO-1 [49–52]. The adherens junction is located below tight junctions and is well connected with tight junctions through a cortical actin–cytoskeleton network [53]. Disruption of tight junctions leads to dissociation of adherens junctions [7, 53]. Furthermore, MMP-9 may cleave E-cadherin [54], which causes dissociation of adherens junctions. Thus, both indirect and direct effects of MMP-9 to E-cadherin may lead to liberation of nectin-1, which becomes more accessible to HSV-1 gD. Notably, increased HSV-1 spread in cells with activated MMP-9 suggests that HIV-activated MMP-9 may not cleave or digest nectin-1.

It is well known that, in HIV/AIDS, HSV reactivation and rapid spread is a common health problem. This could be due to HIV-induced MAPK/NF-κB/MMP-9 activation, which disrupts tight and adherens junctions, liberating nectin-1 and promoting HSV infection and spread. We have shown that HIV tat+gp120-positive CD4^+^ lymphocytes, macrophages and Langerhans cells (LCs) are detected in the oropharyngeal and anogenital epithelia of HIV-infected individuals, including patients receiving antiretroviral therapy [55]. We also have shown that HIV-1 tat and gp120 are secreted into saliva [55]. Furthermore, HIV-infected lymphocytes, macrophages and LCs, cell-free HIV-1 virions and viral DNA/RNA can be detected in the oropharyngeal mucosal epithelium, as well as in the saliva of HIV-positive individuals [56–62]. Thus, oral mucosal epithelium may be exposed to cell-free virions and tat+gp120 proteins from multiple sources, including saliva and circulating immune cells. Interaction of cell-free HIV-1 virions and tat+gp120 proteins with one or more of their receptors, including GalCer, CCR5, CXCR4, α5β1, α5β3, αvβ3 integrins, on the mucosal epithelial cells may induce the activation of MAPKs/NF-κB/MMP-9. This may lead to disruption of tight and adherens junctions and the liberation of nectin-1, promoting HSV-1 infection and spread.

In summary, we have shown that HIV-induced disruption of epithelial junctions could be due to MMP-9 activation through HIV-activated MAPK and NF-κB signalling. MMP-9 may disrupt tight and adherens junctions, liberating nectin-1, which plays a critical role in HSV-1 infection and cell-to-cell spread. Inhibition of MAPK, NF-κB and MMP-9 in the oral and genital epithelium of HIV-infected individuals may reduce the spread of HSV-1 infection and prevent the rapid development of HSV-caused lesions.

## Methods

### Ethics statement

This study was conducted according to the principles expressed in the Declaration of Helsinki and was approved by the Committee on Human Research of the University of California–San Francisco (IRB approval # H8597-30664-03). All human subjects provided written informed consent for the collection of tissue samples. Parents provided informed consent for all minors.

### Establishment of polarized oral epithelial cell monolayers

To establish polarized cells from primary oral epithelial cells, primary keratinocytes were propagated from HIV-negative tonsil tissue samples as described in our previous work [19, 63]. Tonsil epithelial cell lines were grown in culture medium KGM (Lonza) and incubated at 37 °C in a humidified incubator containing 5 % CO_2_. Polarized cells were established in 0.45 µm Transwell two-chamber filter inserts (12-well inserts) as described previously [7, 64, 65]. The polarity of epithelial cells was confirmed by measurement of TER with an epithelial Millicell-ERS voltohmmeter (Millipore Corp.).

### Viruses and viral proteins

Dual-tropic HIV-1_SF33_, R5-tropic HIV-1_SF170_ and X4-tropic HIV-1_92UG029_ were propagated in peripheral blood mononuclear cells as described in our previous work [64, 66]. Molecular clones of the gp120-deficient virus NL4.3-E (HIV-1∆env) were obtained from the NIH AIDS Research and Reference Reagent Program. HIV-1∆env was propagated in HEK293 cells. All HIV stocks were purified using the Amicon Ultra-15 ultracentrifugation filtration system (Millipore). Viruses were titred by p24 concentration using HIV-1 p24 ELISA (ELISA p24) (PerkinElmer) according to the manufacturer’s instructions. Recombinant HIV-1 (BAL strain) wild-type tat and inactive mutant tat proteins were purchased from ImmunoDX (Woburn, MA). Mutant tat was generated by substitution of the basic arginine-rich domain at 49–57 aa and the integrin-binding RGD motif in the C terminus with alanines [55]. HIV-1 (BAL strain) gp120 was provided by the NIH AIDS Research Reagent Program. Heat inactivation of gp120 was performed by incubation at 85 °C for 30 min [67]. HSV-1 (strain F) was grown and titred in Vero cells as described previously [68]. All viruses and proteins were stored in aliquots at 80 °C in the dark before use.

### Treatment of polarized cells with HIV-1 proteins tat and gp120 and cell-free HIV virions

Polarized tonsil epithelial cells were treated with active tat and gp120, inactive mutant tat and heat-inactivated gp120 at a concentration of 10 ng ml^−1^ each. Cells were exposed to dual-tropic HIV-1_SF33_, R5-tropic HIV-1_SF170_ and X4-tropic HIV-1_92UG029_ at a concentration of 20 ng ml^−1^ of p24. Culture medium was changed daily to add fresh virus or proteins [7]. One set of cells was treated with either MAPK inhibitor U0126 (Sigma) at 20 µM or MMP-9 inhibitor I (Calbiochem) at 10 µM. The absence of a toxic effect by virus, tat, gp120 or U0126 was confirmed by MTT cell viability assay (Biotium).

### **Western blot assay to determine MAPK and NF-**κ**B activation**

Polarized tonsil epithelial cells were incubated with HIV tat and/or gp120 recombinant proteins or cell-free HIV virions with or without MAPK inhibitor U0126 at 20 µM. After 5 days, cells were lysed and proteins were separated and transferred to nitrocellulose membranes (GE Healthcare). MAPK activity was measured by detection of phosphorylated and total ERK1/2 protein using phospho-p42/44 MAPK (Erk1/2) (Cell Signaling Technology) and total p42/44 MAPK (Erk1/2) (Cell Signaling Technology). To determine NF-κB activity, we used phospho-Iκbα (Ser32) (14D4) (Cell Signaling Technology), total Iκbα (44D4) rabbit mAb (Cell Signaling Technology), phospho-NF-κB p65 (Se536) (93H1) rabbit mAb (Cell Signaling Technology) and NF-κB p65 (C22B4) rabbit mAb (Cell Signaling Technology). MMP-9 expression was detected using mouse antibody to MMP-9 (Cell Signaling Technology). Protein bands were visualized using the ECL detection system (GE Healthcare). For quantitative analysis of protein bands, films were scanned and saved in TIFF format. The integrated density (pixel intensity over a selected area) of protein bands was quantified by ImageJ with background correction.

### MMP-9 activity by gelatine zymography assay

Cells were incubated with HIV-1 virions and tat+gp120 proteins for 5 days. Supernatants were collected and concentrated. Concentrated supernatants (400 µl) were mixed with SDS sample buffer without a reducing agent, and proteins were subjected to SDS-PAGE in 8 % polyacrylamide gels containing 0.2 % gelatine (v/v). After electrophoresis, the gels were stained with Coomassie Brilliant Blue R250 (Bio-Rad) for 30 min and then de-stained for 1 h in a solution of acetic acid and methanol. Proteolytic activity was evidenced as clear bands (zone of gelatine degradation) against the blue background of stained gelatine. Gels were photographed and saved in TIFF format. Gel images were converted to greyscale mode at DPI 300, and the integrated density of protein bands was quantified by ImageJ with background correction.

### Immunofluorescence assay

To examine tight and adherens junctions, polarized epithelial cells were fixed with 4 % paraformaldehyde for 20 min, permeabilized with 0.05 % Triton X-100 and washed three times with PBS. Cells were then incubated with rabbit mAb occludin (Zymed) and rabbit mAb E-cadherin (CSI) for 1 h at room temperature. Cells were washed and incubated for 25 min with secondary antibody DyLight 488 antimouse IgG (Vector), and cell nuclei were stained with DAPI. Cells were analyzed using a Nikon Eclipse E400 fluorescence microscope.

### HSV-1 cell-to-cell spread in polarized tonsil epithelial cells

To determine cell-to-cell spread of HSV-1 in polarized epithelial cells, a viral plaque assay was used as described previously [7]. Polarized cells were infected with HSV-1 from the basolateral surface at an m.o.i. of 0.01 p.f.u. per cell, and cells were incubated for 2 h at 37 °C. Cells were washed and overlaid with serum-free medium containing 0.5 % methylcellulose from apical and basolateral chambers. After 3 days, cells were fixed and immunostained with mouse antibody to HSV1/2 gD (Santa Cruz Biotechnology). Cells were washed and incubated for 25 min with secondary anti-mouse antibody conjugated with DyLight 488. Cell nuclei were stained with DAPI. Cells were analyzed using a Nikon Eclipse E400 fluorescence microscope (Nikon).

Viral plaques were counted on a minimum of three filter inserts for each experiment, and average plaque numbers were expressed per insert. HSV-1 cell-to-cell spread was evaluated by quantitative analysis of HSV-1-infected plaques. Foci containing five or more infected cells were considered plaques, and cell numbers in each plaque were counted. At least 30 plaques were evaluated for each experimental condition, and the average number of cells per plaque was expressed.

### Statistical analysis

Statistical comparisons were made by two-tailed Student's *t*-test. A *P* value <0.05 was considered significant.
